# Endovascular Embolisation of Visceral Artery Pseudoaneurysms

**DOI:** 10.1155/2014/258954

**Published:** 2014-07-15

**Authors:** Yasir Jamil Khattak, Tariq Alam, Rana Hamid Shoaib, Raza Sayani, Tanveer-ul Haq, Muhammad Awais

**Affiliations:** ^1^Department of Radiology, Aga Khan University Hospital, Karachi, Pakistan; ^2^Department of Radiology, French Medical Institute for Children, Aliabad, Kabul, Afghanistan

## Abstract

*Objective*. To evaluate the technical success, safety, and outcome of endovascular embolization procedure in management of visceral artery pseudoaneurysms. *Materials and Methods*. 46 patients were treated for 53 visceral pseudoaneurysms at our institution. Preliminary diagnostic workup in all cases was performed by contrast enhanced abdominal CT scan and/or duplex ultrasound. In all patients, embolization was performed as per the standard departmental protocol. For data collection, medical records and radiology reports of all patients were retrospectively reviewed. Technical success, safety, and outcome of the procedure were analyzed. *Results*. Out of 46 patients, 13 were females and 33 were males. Mean patient age was 44.79 ± 13.9 years and mean pseudoaneurysm size was 35 ± 19.5 mm. Technical success rate for endovascular visceral pseudoaneurysm coiling was 93.47% (*n* = 43). Complication rate was 6.52% (*n* = 3). Followup was done for a mean duration of 21 ± 1.6 months (0.5–69 months). Complete resolution of symptoms or improvement in clinical condition was seen in 36 patients (80%) out of those 45 in whom procedure was technically successful. *Conclusion*. Results of embolization of visceral artery pseudoaneurysms with coils at our center showed high success rate and good short term outcome.

## 1. Introduction

Visceral arteries include arteries of the splanchnic circulation and the renal arteries [1]. The pseudoaneurysms of visceral arteries (VPAs) are uncommon and attributed to degeneration of the vessel wall mostly due to infections and adjacent inflammation, trauma, and iatrogenic causes [2]. Hemorrhage due to rupture of these pseudoaneurysms is a rare but often life threatening complication which manifests as intra-abdominal or retroperitoneal bleeding and requires emergency treatment [3, 4].

Using digital subtraction angiography the bleeding site can be evaluated followed by embolization of the bleeding vessel or pseudoaneurysm employing superselective catheterization technique [5, 6].

To the best of our knowledge there is no published data available from the developing world regarding clinical presentation, procedural results, and clinical outcome of endovascular management of visceral artery pseudoaneurysms. This study was hence carried out to present details of our initial experience with the procedure at a tertiary care hospital in a third world country.

## 2. Materials and Methods 

This cross-sectional study was carried out at radiology department of a tertiary care hospital in third world country. The study was performed in accordance with the declaration of World Medical Association Declaration of Helsinki. The study was exempted from formal ethical approval as per the institution's policy on retrospective studies and the requirement of informed consent was waived. Data of patients was collected from July 2008 to December 2013. We included all patients who underwent endovascular coiling procedure for visceral artery pseudoaneurysms. A total of 46 patients were found to have visceral artery pseudoaneurysms during the study period.

The patients were referred for treatment to our interventional radiology section from clinical departments of our hospital and from other institutions after being diagnosed to have pseudoaneurysm by contrast enhanced abdominal CT scan or duplex ultrasound examination.

Medical records and images were scrutinized to gather data regarding age, sex, clinical presentation including the symptoms, location, number, and size of aneurysms, technical success, complications, and outcome of the embolization procedures.

**Figure 1:**
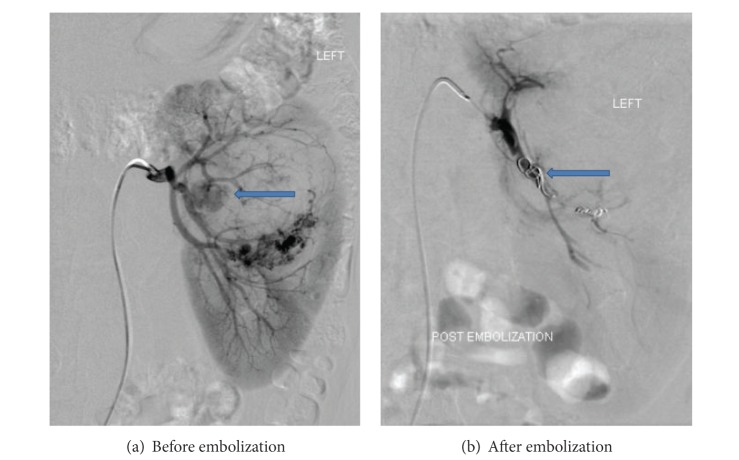
Digital subtraction angiogram. (a) Arrow pointing to a large pseudoaneurysm arising from segmental branch of left renal artery supplying the interpolar region. (b) Arrow pointing to a platinum coil deployed in the segmental branch of left renal artery with successful exclusion of pseudoaneurysm.

**Figure 2:**
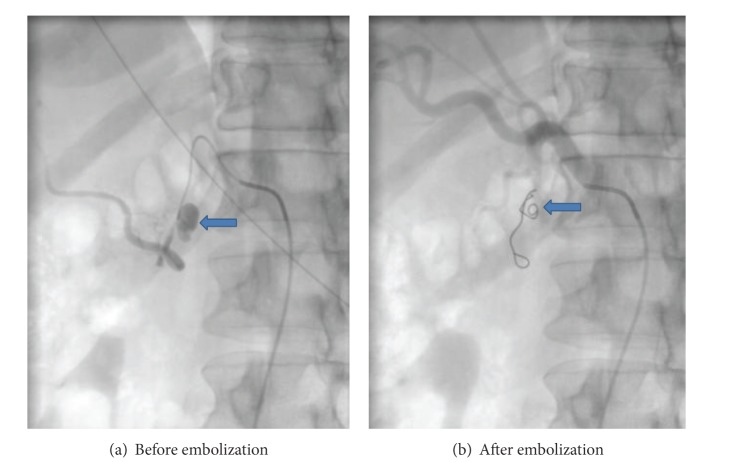
(a) Arrow pointing to pseudoaneurysm arising from branch of gastroduodenal artery. (b) Arrow pointing to platinum coil placed in gastroduodenal artery with successful exclusion of pseudoaneurysm.

Informed consent for the embolization procedure was taken from all patients or their immediate attendants. Embolization was carried out by trained interventional radiologists in dedicated interventional radiology suite on a flat panel monoplane digital subtraction angiography machine (Axiom-Artis; Siemens Medical Systems, Erlangen, Germany). Majority of the cases (30 of 46) were performed under local anesthesia. Femoral artery was punctured for vascular access and a 4 or 5Fr access sheath was placed. Either 4Fr or 5Fr renal double curve catheter (Cordis; Johnson & Johnson, Miami, FL), Sidewinder Simmons, Sim 1 (Cordis; Johnson & Johnsons, Miami, FL), or a Cobra, C1 angiographic catheter (Cook; Bloomington, IN), was advanced over a 0.035 inch guide wire. In cases where there was tortuosity of the vessels or superselective catheterization was required, a microcatheter (Progreat; Terumo, Tokyo, Japan) was used. It was coaxially taken as far as possible, proximal to the aneurysm. Platinum coils were deployed proximally to the aneurysm sac to block the inflow vessel to completely exclude the aneurysm in cases of end arteries (Figures 1 and 2). Outflow vessels were also coiled wherever required as in cases of collateral flow. Technical success was considered as total occlusion of the vascularity of lesion or aneurysmal sac and cessation of hemorrhage seen on postprocedural angiography. Patients were followed after procedure and if required reimaging was done via Doppler ultrasonography or contrast enhanced CT scan. Ultrasound diagnostic equipment (Xario; Toshiba Medical Systems, Tokyo, Japan) was used for performing Doppler examination using 3.6 Mhz convex probe (Xario PVT-674BT; Toshiba Medical Systems, Tokyo, Japan) and/or 7.5 Mhz linear probe (Xario PLT-704SBT; Toshiba Medical Systems, Tokyo, Japan). All CT scans were performed on 64- or 640-slice Multidetector CT (Aquilion; Toshiba Medical Systems, Tokyo, Japan).

## 3. Results

During the study period, 46 patients, 13 females and 33 males, were treated for 53 VPAs. Mean patient age was 44.79 ± 13.90 years (range: 10–77 years). Pseudoaneurysm size ranged from 2 to 69 mm (mean size: 32 ± 21.3 mm). Most common pseudoaneurysm site was renal artery, 23 out of 53 (43.39%), followed by hepatic artery, 14 out of 53 (26.41%). Anatomical distribution, number, and size of pseudoaneurysms are outlined in Table 1.

Four renal artery pseudoaneurysms were identified in a patient, which were filling from different subsegmental arteries; all were cannulated selectively and subsequently embolized. Twelve renal artery pseudoaneurysms were secondary to percutaneous nephrolithotomy (PCNL), two due to postpercutaneous nephrostomy (PCN) insertion and one as a complication of renal biopsy. In four patients renal artery pseudoaneurysms were associated with angiomyolipomas.

**Figure 3:**
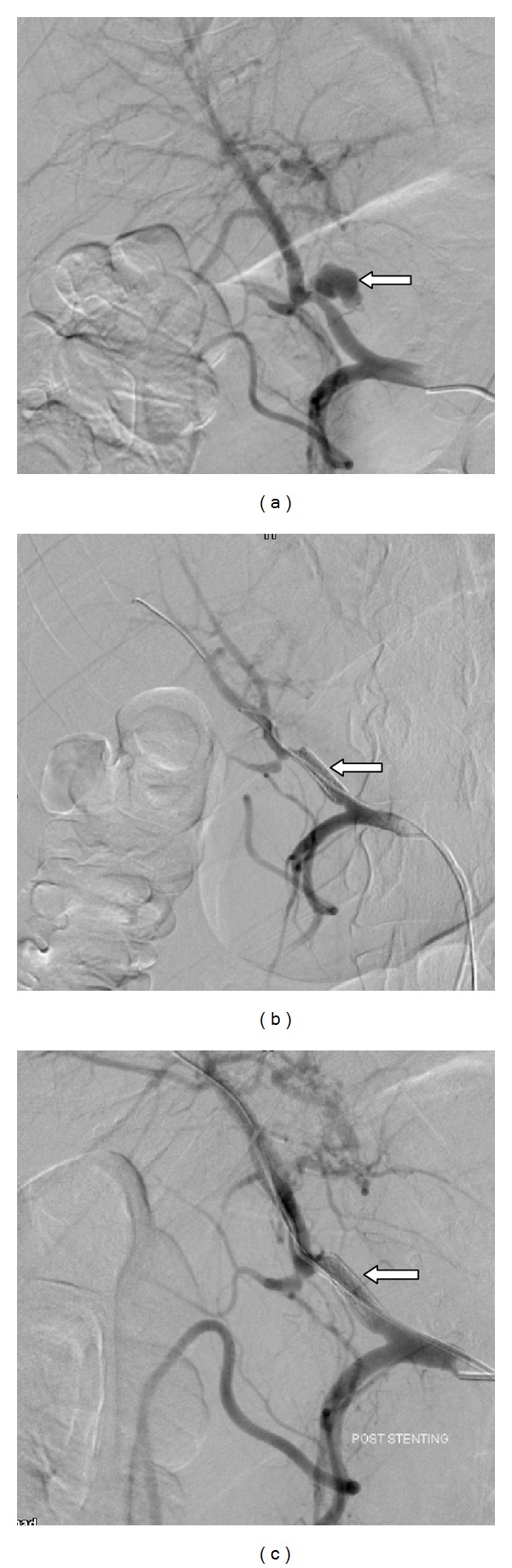
(a) Pseudoaneurysm arising from left hepatic artery (arrow in Figure 3(a)). (b) Covered stent placed across the site of pseudoaneurysm (arrow in Figure 3(b)). (c) Postcovered stent placement angiogram shows complete exclusion of pseudoaneurysm (arrow in Figure 3(c)).

In one patient hepatic artery pseudoaneurysm was mycotic in nature due to adjacent liver abscess. Ten patients developed pseudoaneurysms secondary to liver lacerations following road traffic accidents (Figures 3(a), 3(b), and 3(c)). Another young patient who was a known case of embryonal sarcoma of liver developed multiple pseudoaneurysms within the liver mass.

There were three cases of visceral artery pseudoaneurysm secondary to hepatobiliary interventions, two following laparoscopic cholecystectomy (hepatic artery and cystic artery) and one patient developed pseudoaneurysm of hepatic artery secondary to biliary drain placement. This patient had history of anastomotic stricture following hepaticojejunostomy and underwent cholangioplasty on multiple occasions.

Of the 46 patients, technical success was achieved in 43 patients (93.47%) with preservation of native circulation. In two patients embolization was not successful and the reason of failure was difficult catheterization of the supplying artery, due to complex anatomy and vascular spasm. Both patients were subsequently managed by surgery. One patient had a large pseudoaneurysm of the celiac artery which ruptured during covered stent placement.

A common cause of visceral artery pseudoaneurysm in this series of patients was acute pancreatitis. There were three cases with gastroduodenal artery pseudoaneurysm amongst which two had history of acute pancreatitis. One patient with middle colic artery pseudoaneurysm had history of grade IV pancreatic transaction due to abdominal trauma during a road traffic accident. Another young patient with severe necrotizing pancreatitis developed pseudoaneurysm of left colic artery. Three patients had pseudoaneurysms arising from the splenic artery amongst which two patients had recent history pancreatitis. In one patient the cause was unknown. Two cases with SMA and one with pancreaticoduodenal artery pseudoaneurysms also had pancreatitis as the causative factor. There was one case of IMA (inferior mesenteric artery) pseudoaneurysm for which the cause could not be elucidated.

Complete resolution of symptoms or improvement in clinical condition was seen in 41 patients (91.11%) out of 45 in whom the procedure was technically successful. Six (13.04%) patients required second session of embolization or surgical intervention. Two of these had renal artery pseudoaneurysms associated with angiomyolipomas. One patient had hepatic artery pseudoaneurysm. One patient with embryonal sarcoma of liver had multiple pseudoaneurysms of hepatic artery and required a second session of embolization. One case of necrotizing pancreatitis developed small pseudoaneurysm in close proximity to the previously embolized branch of the middle colic artery. One patient with celiac artery pseudoaneurysm required second session of embolization followed by surgery due to intraprocedural complication.

Procedure related complication rate was 6.52% (3 patients out of 46). In one patient with splenic artery pseudoaneurysm, a small infarct was identified in spleen on followup CT examination. However, patient remained stable and was managed conservatively. In another patient with renal artery pseudoaneurysm, there was dislodgement of coil in the distal profunda femoris artery. However, no significant obstruction to flow was identified. One patient with a large celiac artery became tachycardiac and hypotensive {heart rate 170/min and blood pressure (BP): 60/40 mmHg} during the procedure just before placing the covered stent. Rupture of the aneurysm was suspected which was confirmed with angiogram. Rapid embolization was performed with covered stent. Angiogram showed exclusion of aneurysm; however, patient had low blood pressure and pulse with no spontaneous breathing. CPR was performed which went on for more than 40 minutes. Vitals reverted back and patient was shifted to OR. A second angiogram was performed in the OR as the pressures were still dropping to exclude a leaking pseudoaneurysm. Endoleak was noted from the covered stent so a decision was made to perform laparotomy. During the surgery the patient went into DIC and could not be revived.

Followup was done for a mean duration of 21 ± 1.6 months (range: 0.5–69 months). Six patients were lost to follow up. None of the cases showed puncture site complications, large infarcts, postembolization syndrome, or renal abscess in the mean 21-month followup period. Three patients expired. Amongst these three one case was that of ruptured celiac artery pseudoaneurysm prior to deployment of covered stent. The second patient had necrotizing pancreatitis. There was active extravasation from the splenic artery with pseudoaneurysm formation which was successfully angioembolized with platinum coils; however, the patient had already gone into state of DIC by this time and developed profuse bleeding from multiple sites. Attempts made to resuscitate, however, were not successful. The third case was that of acute pancreatitis with pancreaticocolocutaneous fistula. During his hospital stay he developed multiple drug resistant organism infection. He was continuously kept on breathing support. The patient already had one session of successful angioembolization. In ICU he developed frank bleeding from the fistula in the epigastrium. Patient was taken to OR and attempts were made to control bleeding. Meanwhile the patient became asystolic. Attempts made to resuscitate the patient, however, could not be revived.

## 4. Discussion 

In the past, surgery has been the method of treatment of both ruptured and unruptured aneurysms and pseudoaneurysms [7, 8]. With development of newer interventional techniques and increasing experience of interventional radiologists, traditional concepts of treatment have changed [9, 10].

Endovascular management is safe and effective with fewer complications, shorter hospital stay, and faster recovery [11–13]. The various methods for endovascular treatment include placement of coils, deployment of covered stents, injecting polyvinyl alcohol particles, gelfoam or glue, and endoluminal thrombin injection [9, 14–17]. In our series platinum coils of various lengths and diameters were used for endovascular treatment. Rare technical complications of coiling include parent artery occlusion, aneurysm perforation, coil migration, and aneurysm recurrence [18, 19].

Renal artery pseudoaneurysms were most common (43.39%) and most of these were related to iatrogenic vascular injury (22.64%, *n* = 12) especially due to percutaneous nephrolithotomy. Our technical success rate was 93.47% which is quite similar and comparable to that reported by Sethi et al., Piffaretti et al., and Zhu et al. [5, 20, 21].

Ruptured celiac artery pseudoaneurysm during covered stent placement was the only major complication in this series of patients. Minor procedural complications in our series were 6.6%. For both patients no active management was required and both were treated conservatively. Our complication rate is much better than the complication rate of 37.5 reported by Piffaretti et al. [20].

A total of 10 out of 46 patients (21.7%) received second session of endovascular embolization and/or surgical treatment as salvage procedure after the first procedure failed. In two out of these ten patients, first session of embolization was not technically successful due to complex vascular anatomy and vascular spasm. In four patients, reperfusion was the indication for subsequent session of endovascular or surgical treatment. One patient amongst these four had post-PCNL hematuria and pseudoaneurysm was successfully embolized in the first session but a repeat angiogram was later carried out because of persistent hematuria which showed contrast extravasation from a different subsegmental branch which was then superselectively catheterized and occluded successfully with a microcoil. Another patient had bilateral angiomyolipomas (AML); in this patient embolization was repeated three days later after the first session; however, hematuria persisted and nephrectomy had to be performed. Similarly in the other patient with AML, the small feeding vessel filling the pseudoaneurysm could not be successfully cannulated and therefore the segmental branch was embolized using PVA particles; however, hematuria could not be controlled and patient later underwent nephrectomy. One patient had a large pseudoaneurysm of the distal main hepatic artery which was compressing the main portal vein and common bile duct. The pseudoaneurysm was embolized; however, the patient presented three days later with recurrent hematemesis and melena and was managed by surgical ligation. On one occasion where an endoleak was suspected following covered stent placement angiography had to be performed just prior to laparotomy in the OR. One patient with history of pancreatic transaction underwent multiple angiograms to evaluate cause of dropping hemoglobin level. Mesenteric angiogram revealed active extravasation of contrast from branches of middle colic artery with pseudoaneurysm formation which was successfully embolized with platinum coils and PVA particles. The patient had multiple episodes of melena few days later, on account of which GI bleed was suspected and another angiogram was done which turned out to be negative. A CT was performed few days later which showed a large pancreataic pseudocyst. Since the hemoglobin levels were continuously dropping, a third angiogram was carried out. This time a small pseudoaneurysm was identified lying in close proximity to the previously embolized branch of the right colic artery.

The reintervention rate of 10.86% in our series is quite similar and comparable to that of Spiliopoulos et al. and Huang et al. [16, 22].

There was one procedure related mortality where a celiac artery pseudoaneurysm ruptured prior to deployment of a covered stent. One patient in our series had posttraumatic liver laceration and hepatic artery pseudoaneurysm on angiography which was successfully embolized but patient died due to disseminated intravascular coagulation and other multiorgan injuries. One patient with pancreaticocolocutaneous fistula developed multidrug resistant organism infection during his hospital stay. The patient already had one session of successful angioembolization of left colic artery. In ICU he developed frank bleeding from the fistula in the epigastrium. Patient was taken to OR and attempts were made to control bleeding. Meanwhile the patient became asystolic. Attempts made to resuscitate the patient, however, could not be revived.

We would like to mention a few limitations of our study. First, being a retrospective review, the study has inherent deficiencies, especially while recording the fine technical details of procedure and clinical examination of patients at presentation as well as at followups. Secondly since it is only our initial experience, the number of cases is also small. Lastly, the outcome measure was based on clinical criteria and followup angiograms were not performed if patient was clinically improving. Despite these limitations, this study is one of the first reported series of visceral artery aneurysm embolization from a third world country and in our opinion would serve as a baseline for monitoring further regional progress. Larger prospective studies are nevertheless recommended for even better evaluation and more detailed analysis of determinants of complications and outcome. The procedural success rates, safety, and eventually patient outcome are expected to improve further with increasing experience of interventional radiologists.

## 5. Conclusion

Results of endovascular pseudoaneurysm embolization with coils at our center showed high technical success rate and good short term clinical outcome.

## Figures and Tables

**Table 1:** Number, size, and anatomical distribution of the aneurysms.

Artery of origin	Number of aneurysms	Size range
Total	53	
Renal	23	Range: 4.8–69 mm
Hepatic	14	Range: 7–44 mm
SMA	2	Range: 28–36 mm
Splenic	3	Range: 16–55 mm
IMA	1	Range: 15 mm
Cystic	1	Range: 19 mm
Celiac	2	Range: 43–45 mm
Gastroduodenal	3	Range: 11–13 mm
Pancreaticoduodenal	1	Range: 8 mm
Left colic	2	Range: 6–8.5 mm
Middle colic	1	Range: 4.5 mm
